# Privacy-Enhanced and Multifunctional Health Data Aggregation under Differential Privacy Guarantees

**DOI:** 10.3390/s16091463

**Published:** 2016-09-10

**Authors:** Hao Ren, Hongwei Li, Xiaohui Liang, Shibo He, Yuanshun Dai, Lian Zhao

**Affiliations:** 1 School of Computer Science and Engineering, University of Electronic Science and Technology of China, Chengdu 611731, China; jingsboy@126.com (H.R.); uestcdaiys@gmail.com (Y.D.); 2 State Key Laboratory of Information Security, Institute of Information Engineering, Chinese Academy of Sciences, Beijing 100093, China; 3 Department of Computer Science, University of Massachusetts at Boston, MA 02125, USA; Xiaohui.Liang@umb.edu; 4 State Key Laboratory of Industrial Control Technology, Zhejiang University, Hangzhou 310027, China; s18he@iipc.zju.edu.cn; 5 Department of Electrical and Computer Engineering, Ryerson University, Toronto, ON M5B 2K3, Canada; lzhao@ee.ryerson.ca

**Keywords:** cloud-assisted WBANs, privacy-enhanced, multifunctional aggregation, health data, fault tolerance, differential privacy

## Abstract

With the rapid growth of the health data scale, the limited storage and computation resources of wireless body area sensor networks (WBANs) is becoming a barrier to their development. Therefore, outsourcing the encrypted health data to the cloud has been an appealing strategy. However, date aggregation will become difficult. Some recently-proposed schemes try to address this problem. However, there are still some functions and privacy issues that are not discussed. In this paper, we propose a privacy-enhanced and multifunctional health data aggregation scheme (PMHA-DP) under differential privacy. Specifically, we achieve a new aggregation function, weighted average (WAAS), and design a privacy-enhanced aggregation scheme (PAAS) to protect the aggregated data from cloud servers. Besides, a histogram aggregation scheme with high accuracy is proposed. PMHA-DP supports fault tolerance while preserving data privacy. The performance evaluation shows that the proposal leads to less communication overhead than the existing one.

## 1. Introduction

Recently, many information techniques have been utilized for healthcare systems [1] to reduce the expenses and boost the efficiency of medical services. Specifically, the application of wireless body area networks (WBANs) [2] is a promising technique, which has a huge potential to revolutionize the future of healthcare monitoring by diagnosing many life-threatening diseases and providing real-time patient monitoring [3]. WBANs can continuously monitor patient’s physiological attributes, such as blood pressure, body temperature, electrocardiograph (ECG), electroencephalogram (EEG) [4], and so on. Usually, all of the detected data are gathered by the user’s personal digital assistant (PDA) or smartphone. Hence, aggregating and analyzing this sensitive health information is crucial for medical institutions.

In the era of big data, health data aggregation is the basic work of medical big data analysis. Actually, both spatial and temporal aggregation services are widely applied in many healthcare scenarios. For instance, in an elderly community, there are some patients who suffer from hypertension. All of these patients may be equipped with body area sensors to monitor their blood pressure. The collected data of each patient are uploaded to the authorized hospital via smart devices. Therefore, the hospital can obtain each user’s maximum/minimum blood pressure over the past day. If one patient’s blood pressure is abnormal, the doctor may ask the patient to come to the hospital to have a further treatment. It is a typical temporal aggregation [5] case, which helps hospital provide better healthcare service. For a medicine research center and health bureau, the spatial aggregate statistics [6] (e.g., the average blood pressure of users in a specific community) may be much more useful than temporal aggregation.

However, with the rapidly increasing amount of health information, the cost of providing e-health services is becoming high for some hospitals and medical research institutions. In addition, the traditional WBANs have limited computation and storage resources, which have difficulty satisfying the needs of practical applications. Therefore, outsourcing large-scale health data to the cloud is a cost-efficient choice to release the burdens of data storage and data management. Therefore, in this paper, we leverage the cloud-assisted WBANs to accomplish the mission of storing and processing health data. Unfortunately, the original system model of cloud computing may suffer from the problems of data security and user privacy. Because the cloud server is honest-but-curious, it may reveal the content of personal health information. For instance, a patient’s living and eating habits can be reflected by his or her ECG. Thus, the health data should be protected from malicious entities. A direct solution to preserve the privacy and integrity of health data is to let the data owners encrypt them before outsourcing. However, conventional encryption schemes make data aggregation computation harder to run. Thus, many privacy-preserving schemes are proposed to address this problem, such as [7,8,9]. However, these schemes only calculate summation aggregation or additive aggregation [10]. Recently, Han et al. [6] proposed a scheme to achieve additive and non-additive aggregation simultaneously. However, some functions are still not discussed, such as the weighted average. Besides, the histogram aggregation is also not investigated thoroughly in [6]. Furthermore, the sum of all of the users’ health data may be disclosed by cloud servers in Han et al.’s scheme [6].

In this paper, to address the above problems, we propose a privacy-enhanced and multifunctional health data aggregation scheme under differential privacy guarantees (PMHA-DP). Specifically, the main contributions of this paper can be summarized as follows:

- First, we propose a basic average aggregation scheme (BAAS) by utilizing the Boneh–Goh–Nissim cryptosystem. Note that, in some scenarios, the data analysts would prefer to acquire the weighted average; because, the weighted average can be more objective to reflect the overall state of all users. Thus, we propose a privacy-preserving weighted average scheme (WAAS) to meet the above requirement. To the best of our knowledge, this paper is the first to discuss the weighted average aggregation. Besides, the final results of both schemes are protected by differential privacy mechanisms [11].
- Second, we provide a privacy-enhanced average aggregation scheme (PAAS) to protect the sum of all of the gathered health data. In [6], one of the working cloud servers is able to obtain the plaintext of the sum. If this server is compromised, it may leak the information to some malicious entities. Therefore, we design a protocol with additional private keys to hide the aggregated data. PAAS hides the sum of the dataset from the cloud servers. It further protects the users’ privacy.
- Third, histogram aggregation is well studied in this paper. We leverage the hierarchical method [12] for histogram (HMH). Then, we add Laplace noise [13] to the query result. Moreover, we also leverage the post-processing technique [12] to boost the accuracy of the released answer.
- Finally, we conduct real experiments and compare PMHA-DP with the other scheme [6]. The comparison results show that our non-additive aggregation scheme (NAS) and PAAS lead to less communication overhead than that of another scheme called ${\mathrm{MHDA}}^{⊕}$ [6]. Besides, PAAS enhances the data privacy with acceptable computational overhead. Moreover, we give a security analysis to show that the proposed scheme preserves data privacy under the given strong adversary model. More importantly, all of the proposed aggregation protocols support fault tolerance.

The remainder of this paper is organized as follows. In Section 2, the system model of PMHA-DP, the adversary model and the security requirements are formalized. We recall the Boneh–Goh–Nissim cryptosystem and differential privacy in Section 3. In Section 4, we propose our scheme. This is followed by the security, privacy analysis and performance evaluation in Section 5 and Section 6, respectively. In Section 7, we give some further discussions on four vital topics. The related work is introduced in Section 8. Finally, we conclude this paper in Section 9.

## 2. Problem Statement

In this section, we will introduce the system model, adversary model, security and privacy requirements of PMHA-DP to formalize the research problems.

### 2.1. System Model

**Figure 1:**
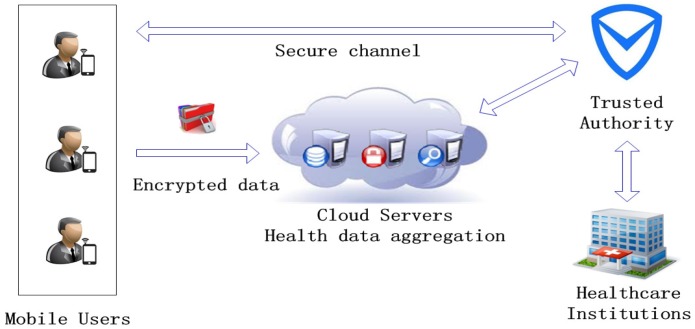
System model of privacy-enhanced and multifunctional health data aggregation scheme (PMHA-DP).

As shown in Figure 1, the system model of PMHA-DP is composed of four entities: mobile users, cloud servers, trusted authority and healthcare institutions. Each entity is introduced as follows.

- Mobile users (MUs): MUs are the data providers of the cloud-assisted WBAN system, which are denoted as $U=\{U_{1},U_{2},...,U_{k}\}$. Specifically, $U_{i}$ is equipped with some body area sensors to monitor different types of health data. Then, the original health data collected by sensors will be stored in $U_{i}$’s smartphone or PDA. For privacy consideration, MUs encrypt the data using the smartphone before reporting them to the cloud servers. Furthermore, MUs report the personal health data according to the aggregation protocols formulated by the trusted authority.
- Cloud servers (CSs): CSs are a group of public cloud servers denoted as $S=\{S_{1},S_{2},...,S_{n}\}$. In PMHA-DP, multiple servers are necessary for executing the aggregation missions and supporting fault tolerance. A large volume of health data is stored in CSs. The aggregation result will be delivered to the trusted authority instead of healthcare institutions directly. According to the practice, we assume that all of the CSs are honest-but-curious. CSs store and process data honestly, but they may be also curious about individual user’s health data. Thus, CSs only store the ciphertexts of health data received from MUs. Since CSs are powerful, we assume that a strong adversary can compromise or paralyze no more than l=⌈n/2⌉−1 cloud servers.
- Trusted authority (TA): TA is a powerful data center, which is responsible for assigning aggregation tasks and key management. TA receives different aggregation requests from healthcare institutions, then it bootstraps the whole system. In the initialization phase, TA first generates secret keys and US certificates for each registered user. Besides, keys and certificates are distributed through a secure channel. Meanwhile, TA also generates private keys for cloud servers. If TA wants some statistical information of the health dataset, it will make l+1 cloud servers work together to aggregate and decrypt the data. Then, the system randomly selects one of the working cloud servers to send the statistics to TA. At last, TA will calculate the final result and adds noise to it by utilizing differential privacy mechanisms. TA is the only globally-trusted entity of the whole system.
- Healthcare institutions (HIs): HIs represent the organizations (i.e., certified hospital, medicine research center, health departments, etc.) that are interested in the statistical information of a large volume of health data. HIs obtain this information by sending specific requests to TA, and TA returns the final result to HIs.

In order to convince us that the system model is practical, we make a further discussion. Differential private techniques are firstly designed to resist the differential attacks. As the development of differential privacy, data sanitizing schemes are proposed. Those schemes add a little bit of noise to the users’ local data to protect the sensitive information. However, those techniques cannot hide all of the personal information. For instance, a user adds a little noise to his/her body temperature (suppose the original value is 37). The noisy value may be quite close to the original value, such as 37.5. If the data are not encrypted, the public cloud can deduce the distribution and some other statistical information of the dataset. The mathematical expectation of the permutated dataset is the same as the original dataset. Therefore, the cloud server can infer that the dataset must be the users’ temperatures if the mathematical expectation is about 37. Since the ciphertext contains no statistical information, all of that sensitive information can be protected against the public cloud if all users’ data are encrypted. In order to resist differential attack, we add noises to the final aggregation results instead of encrypting the data (again) and sending the encrypted data to HI. If we choose to encrypt the results again without permutation, HI can deduce some individual records’ by asking legitimate queries. Thus, it is necessary to release the result under differential privacy.

### 2.2. Adversary Model

In PMHA-DP, a strong adversary Adv is considered. Adv may launch the following attacks:

- Adv may eavesdrop on communication flows.
- Adv may compromise some users directly.
- Adv may compromise less than l=⌈n/2⌉−1 CSs to breach users’ privacy.
- In our privacy-enhanced health data aggregation scheme, Adv may compromise all of the l+1 working cloud servers and obtain the sum of all users’ private data.
- Adv may launch differential attacks on TA (e.g., Adv may deduce the newly-added users’ data by asking TA legitimate queries).

### 2.3. Security and Privacy Requirements

The adversary Adv would like to reveal as much of the users’ personal private information as possible. Therefore, the designed data aggregation system should resist Adv’s attacks. Specifically, the scheme must satisfy the following security and privacy requirements:

- Adv cannot reveal users’ private health data, even if the communication flows are intercepted.
- Adv cannot reveal the uncompromised users’ private health data, even if some users are compromised directly.
- Adv cannot reveal users’ private health data, even if *l* cloud servers are compromised.
- Adv cannot obtain the sum of all of the users’ private data, even if all of the l+1 working cloud servers are compromised.
- Adv cannot deduce any individual user’s health data by launching differential attacks on TA.

## 3. Preliminaries

In this section, we will briefly present the concept of differential privacy and the cryptographic building block that PMHA-DP builds on. At last, we give a further discussion on the data type and differential privacy mechanism.

### 3.1. Boneh–Goh–Nissim Cryptosystem

Boneh et al. [14] presented a homomorphic public key encryption scheme based on finite groups of composite order that support a bilinear map. Their system supports arbitrary additions and one multiplication on encrypted data. Due to those homomorphic features, it is often used to achieve privacy-preserving data aggregation tasks. Here, we introduce the three algorithms making up the system.

- KeyGen(τ): Given a security parameter $\tau \in Z^{+}$, the system runs Gen(τ) to acquire a tuple $\left(p,q,G,G_{1},e\right)$. Here, G and $G_{1}$ are two cyclic groups of order n=pq. In addition, $e:G\times G\to G_{1}$ is a bilinear map [15]. Randomly pick two generators g,u∈G, and set $h=u^{q}$. Then, *h* is a random generator of the subgroup of G of order *p*. The public key is $PK=(n,G,G_{1},e,g,h)$. The private key is SK=p.
- Encrypt(PK,m): Let m∈{0,1,...,T} represent a message, and *T* (T≪q) is the upper bound of the message space. To encrypt a message *m* using public key PK, the user picks a random number *r* $\left(r\in Z_{n}\right)$ and calculates the ciphertext as $C=g^{m}h^{r}\in G$.
- Decrypt(SK,C): The system decrypts ciphertext *C* with private key SK=p through computing $C^{p}={\left(g^{m}h^{r}\right)}^{p}={\left(g^{p}\right)}^{m}$. Let $\hat{g}=g^{p}$. Then, the system computes the discrete logarithm of $C^{p}$ base $\hat{g}$ to recover *m*. The computation takes the expected time $O(\sqrt{T})$ using Pollard’s lambda method [16].

Here, we introduce the two homomorphic properties of the Boneh–Goh–Nissim cryptosystem.

1. Firstly, the system is clearly additively homomorphic. Given any two ciphertexts $C_{1},C_{2}\in G$ of messages $m_{1},m_{2}\in \left\{0,1,...,T\right\}$, respectively, one can obtain the encryption of $m_{1}+m_{2}$ by computing the product $C=C_{1}C_{2}$.
2. Secondly, one can multiply two encrypted messages once using the bilinear map to acquire the product of two messages. Let $g_{1}=e\left(g,g\right)$, $h_{1}=e\left(g,h\right)$, and set $h=g^{\alpha q}$ where α∈Z is unknown. Suppose that the two given ciphertexts are $C_{1}=g^{m_{1}}h^{r_{1}}\in G$ and $C_{2}=g^{m_{2}}h^{r_{2}}\in G$. Then, we have:
  (1) $$\begin{array}{cc} C=e\left(C_{1},C_{2}\right)=e\left(g^{m_{1}}h^{r_{1}},g^{m_{2}}h^{r_{2}}\right) & =g_{1}^{m_{1}m_{2}}h_{1}^{m_{1}r_{2}+m_{2}r_{1}+\alpha qr_{1}r_{2}}\\ & =g_{1}^{m_{1}m_{2}}h_{1}^{\bar{r}}\in G_{1} \end{array}$$
  where $\bar{r}=m_{1}r_{2}+m_{2}r_{1}+\alpha qr_{1}r_{2}$, and $\bar{r}\in Z_{n}$. Thus, *C* is the ciphertext of $m_{1}m_{2}$, and the recovery of $m_{1}m_{2}$ is similar to $C_{1},C_{2}$. Furthermore, the system is still additively homomorphic in $G_{1}$.

### 3.2. Differential Privacy

Differential privacy [11] was first proposed by Dwork. Informally, if an algorithm is not sensitive to small changes in the input, then it may be differentially private. The idea of a differential privacy protection model is derived from a very simple observation: when the dataset *D* contains individual Alice, let *f* be arbitrary query operation on *D* (such as count, sum, average, median or other range queries, etc.); the results obtained are f(D). If the result of the query is still f(D) when Alice’s record is deleted, you can indicate that Alice’s privacy is protected from the differential attack. Differential privacy is to ensure that any operation on a single record (e.g., add, remove or change a record) cannot impact the final result of the query. In other words, there are two almost identical sets of data (only one record is different); the probability of getting the same result from the same query that is operated on the two datasets is close to one.

Formally, for any given database instance *D*, let nbrs(D) denote the set of neighboring databases differing from *D* by at most one record; i.e., if $D^{\prime }\in nbrs\left(D\right)$, then $|\left(D-D^{\prime }\right)\cup \left(D^{\prime }-D\right)|=1$. Let function Range(A) be the output range of the random algorithm A. Then, the formal definition is shown below.

**Definition** **1.** *An randomized algorithm A is ϵ-differentially private if for all instances D, any $D^{\prime }\in nbrs\left(D\right)$, and any subset of outputs S⊆Range(A), the following holds:*

(2) $$Pr\left[A\left(D\right)\in S\right]\leq \exp\left(\epsilon \right)\times Pr\left[A\left(D^{\prime }\right)\in S\right],$$

*where ϵ is the privacy budget.*

Different from the encryption-based schemes, differential privacy does not have a security level (e.g., secure under chosen plaintext attack, etc.). The privacy level of the differential private algorithm depends on the privacy budget *ϵ*. It is set by the user; the larger the privacy budget is, the smaller the noise is. Thus, the privacy budget is inversely proportional to the accuracy of the query result.

In this paper, we adopt the Laplace mechanism [13] to design differential privacy algorithms. The Laplace mechanism utilizes the sensitivity of the query function to calibrate the noise scale. The definition of the function sensitivity is shown as below.

**Definition** **2.** *Given a function f:D→R, the sensitivity of f, denoted Δf, is:*

(3) $$\Delta f=\max_{D,D^{\prime }}{\left|f\left(D\right)-f\left(D^{\prime }\right)\right|}_{1}\;\;\;\;s.t.\;\;\;\;D^{\prime }\in nbrs\left(D\right).\;$$

Let Lap(λ) denote the Laplace probability distribution with mean zero and scale *λ*. The Laplace mechanism achieves differential privacy by adding Laplace noise to the output of function *f*.

**Definition** **3.** *Let f be an aggregation function of a database D and Z be a random variable where Z∼Lap(Δf/ϵ). The Laplace mechanism $\tilde{f}$ is defined as:*

(4) $$\tilde{f}\left(D\right)=f\left(D\right)+Z.$$

The randomized algorithm $\tilde{f}$ is *ϵ*-differentially private.

The data type in this paper is numerical. We cannot calculate the statistics of the categorical data under the ciphertext environment. For instance, there exists an attribute recording the favorite sports of the user. In the database, we can use integral numbers to represent the categorical data (e.g., one represents ąřfootballąś, two represents ąřbasketballąś, etc.). However, it is meaningless to calculate the average or summation of the categorical data. Since the aggregation tasks can be assigned to the cloud in any time, the cloud servers and the data should be online. The encrypted data are stored in relational tables. However, the attribute types and the record values are all encrypted or permutated. There is no limit to the size of the data. The user could encrypt some short health message, such as blood pressure, temperature, and so on. Therefore, the expected maximum value of those health data depends on the observation of clinical medicine. For example, a person’s blood pressure is usually less than 140 mmHg. Then, we can use this value to quantify the sensitivity of aggregation functions.

Interactive and non-interactive differential privacy mechanisms are significantly different. The interactive mechanism allows the user to query several times until the privacy budget is consumed. The non-interactive mechanism answers all of the queries at one time. The Laplace mechanism applied in this paper is interactive. Therefore, the proposed differential privacy scheme is interactive.

## 4. Proposed Scheme

In this section, we propose a multifunctional health data additive aggregation scheme and a non-additive aggregation scheme, respectively. “Multifunctional” means that we provide different aggregation functions. It is an important property that reflects the scalability and the practicability of a data aggregation scheme. Our scheme achieves the average, the summation, the median and some other aggregation functions simultaneously. In the first part, we only illustrate the average aggregation as an example of additive aggregation. In the second part, we will discuss various non-additive aggregations, such as min/max, median, histogram, etc., which are widely applied in reality. Furthermore, advanced schemes are also proposed.

### 4.1. Additive Aggregation of Health Data

In this part, we will show the details of the basic average aggregation and weighted average aggregation scheme. Besides, the final results calculated by TA strictly satisfy the differential privacy. Compared with the scheme proposed by Han et al. [6], PMHA-DP is well designed with less noise being added, which improves the accuracy of the final results significantly. In [6], the cloud servers are able to decrypt and learn the sum of health data. However, the statistical information (i.e., sum of the dataset) may be sensitive and should not be disclosed to the public. On addressing the above problem, we propose a new advanced average aggregation protocol.

#### 4.1.1. System Initialization

In the phase of initialization, TA bootstraps the system and generates public and private keys. Firstly, TA runs Gen(τ) to obtain a bilinear map tuple $\left(p,q,G,G_{1},e\right)$. As previously stated in Section 3.1, TA generates the tuple $\left(n,G,G_{1},e,g,h\right)$ by leveraging the Boneh–Goh–Nissim cryptosystem. $h=g^{q}$ is a random generator of the subgroup of G of order *p*, and g∈G is a random generator of G. In addition, TA chooses a one-way hash function $H:{\left\{0,1\right\}}^{*}\to G$. Hence, the public key is $\left(n,G,G_{1},e,g,h,H\right)$, and the private key is *p*. Moreover, the private key SK=p is seen as a shared secret, and each share of *p* is assigned to each working cloud server. For simplicity, TA utilizes Shamir’s [17] secret share scheme. TA first randomly generates a polynomial function $G\left(x\right)=p+a_{1}x+a_{2}x^{2}+...+a_{l}x^{l}$ $s.t.\;\;\;i=\;1,2,...,l.\;\;\;a_{i}\in Z_{n}$, then TA calculates G(j) as $S_{j}$’s private key, where $S_{j}\in S$.

#### 4.1.2. Basic Average Aggregation Scheme

Since we can easily calculate the average of the dataset from its sum, the working CSs firstly compute the sum and deliver it to TA. Then, TA computes the average of dataset. Details are shown as follows.

User generates ciphertext: Mobile user’ health data are detected by body sensors, and the original data are all stored and processed in the user’s smartphone or PDA. The detected data directly reflects the health status of users. Thus, MUs would like to report their health data to the healthcare institutions and acquire health services. However, the health data may leak some sensitive information of individuals. Consequently, users encrypt their data before submitting them to the public cloud. Specifically, each user $U_{i}\in U$ encrypts his or her health data $m_{i,o}\in \left\{0,1,...,T\right\}$ at time point $t_{o}$ through the following three steps:

- Step 1: $U_{i}$ computes the hash value $\theta_{o}=H\left(t_{o}\right)$ at the time point $t_{o}$.
- Step 2: $U_{i}$ encrypts the message $m_{i,o}$ through calculating $C_{i,o}=g^{m_{i,o}}h^{\theta_{o}\cdot r_{i,o}}$, where $r_{i,o}\in Z_{n}^{+}$ is a random number.
- Step 3: $U_{i}$ submits the ciphertext $C_{i,o}$ to one of the working cloud servers.

Privacy-preserving computing of ${\mathrm{Sum}}_{o}$: One of the working cloud servers receives all of the users’ encrypted health data $\left\{C_{1,o},C_{2,o},...,C_{k,o}\right\}$ of message set $\left\{m_{1},m_{2},...,m_{k}\right\}$. According to the privacy requirements, the cloud server needs to compute the encrypted sum without knowing the plaintext of any specific message. By utilizing the homomorphic property of Boneh–Goh–Nissim, we can obtain the encrypted aggregation $Sum_{o}$ as follows:

(5) $$Sum_{o}=\prod_{i=1}^{k}C_{i,o}=\prod_{i=1}^{k}\left(g^{m_{i,o}}\cdot h^{\theta_{o}\cdot r_{i,o}}\right)=g^{\sum_{i=1}^{k}m_{i,o}}\cdot h^{Z},\;$$

where $Z=\sum_{i=1}^{k}\left(\theta_{o}\cdot r_{i,o}\right)\;mod\;p$.

Joint decryption of ${\mathrm{Sum}}_{o}$: Before each aggregation task begins, TA randomly chooses l+1 cloud servers δ⊂S as the working servers. As mentioned above, one of the cloud severs calculates $Sum_{o}$ and sends it to the other *l* working cloud servers. Upon receiving $Sum_{o}$ at time point $t_{o}$, each cloud server first calculates:

(6) $$\gamma_{j}=\prod_{i\in \delta ,i\neq j}\frac{i}{i-j}$$

then computes:

(7) $$d_{j,o}=Sum_{o}^{\gamma_{j}G\left(j\right)}$$

Then, TA selects one of the l+1 servers to gather all of the $d_{j,o}$ computed by each $S_{j}\in \delta$ and calculates:

(8) $$\begin{array}{cc} D_{o} & =\prod_{S_{j}\in \delta }d_{j,o}=\prod_{S_{j}\in \delta }Sum_{o}^{\gamma_{j}G\left(j\right)}\\ & =Sum_{o}^{\sum_{S_{j}\in \delta }\gamma_{j}G\left(j\right)}=Sum_{o}^{p}={\left(g^{\sum_{i=1}^{k}m_{i,o}}\right)}^{p}\cdot {\left(h^{Z}\right)}^{p}\\ & ={\left(g^{p}\right)}^{\sum_{i=1}^{k}m_{i,o}}={\hat{g}}^{\sum_{i=1}^{k}m_{i,o}} \end{array}$$

where $\hat{g}=g^{p}$. According to the Lagrange interpolation polynomial [17], we have:

(9) $$G\left(x\right)=\sum_{j=0}^{l}\left(\prod_{i=0,i\neq j}^{l}\frac{x_{i}-x}{x_{i}-x_{j}}\right)G\left(x_{j}\right)$$

Thus,

(10) $$\sum_{S_{j}\in \delta }\gamma_{j}G\left(j\right)=\sum_{j=0}^{l}\left(\prod_{i=0,i\neq j}^{l}\frac{i-0}{i-j}\right)G\left(j\right)=G\left(0\right)=p$$

Since $m_{i}\in \left\{0,1,2,...,T\right\}$, we have $\sum^{*}=\sum_{i=1}^{k}m_{i,o}\leq \left(k+1\right)T$. The CS can obtain the sum of users’ health data $\sum^{*}$, by computing the discrete logarithm of $D_{o}$ based on $\hat{g}$ in expected time $O(\sqrt{(k+1)T})$. At last, the CS sends $\sum^{*}$ to TA.

Result release under differential privacy: Different from scheme proposed in [6], CS cannot release any data to the analyst directly. CS must send the intermediate result to TA first. TA is responsible for computing the final result and releasing it under differential privacy. The details of the computation and proof of differential privacy are shown as follows.

Once TA receives the sum of dataset $\sum^{*}$, TA can get the average value by simply calculating $M=\frac{\sum^{*}}{k}$. In order to ensure differential privacy, we add noise to *M* by leveraging the Laplace mechanism. Let Z be a random variable, and $Z∼Lap(\frac{T}{\epsilon (k-1)})$. Here, Z is the Laplace noise, which will be added to *M*. Then, TA simply computes $\tilde{M}=M+Z$. Finally, $\tilde{M}$ is returned to the data analyst (HIs) as the final result. We can assert that the released result $\tilde{M}$ is *ϵ*-differentially private. The mainly challenge is the proof of the differential privacy. The formal proof of differential privacy is given in Section 5.

#### 4.1.3. Weighted Average Aggregation Scheme

In a real data aggregation scenario, each data source is weighted by its reliability. For instance, a data provider may submit abnormal data. Then, the weight of this provider should be lower than the normal ones. In WAAS, the *i*-th provider’s weight is denoted by a non-integral number $w_{i}\in \left\{0,1,...,T\right\}$. All of the weights of the providers are stored in TA represented as a weight vector $W=(w_{1},w_{2},...,w_{k})$. Moreover, the sum of all of the weights is $\sum_{weight}$, which is also kept by the TA. In addition, the plaintext of mobile users’ data also could be represented by a vector $M=(m_{1},m_{2},...,m_{k})$. We can regard the weight vector W as a batch of messages, which has no difference between the users’ data vector W. Therefore, the weighted sum of the dataset is the inner product of M and W. Similar to the basic aggregation scheme, we first calculate weighted sum $\sum^{*}=W\cdot M$. Details are shown as follows.

User generates ciphertext: Similar to the basic scheme, the data should be encrypted before sending to the cloud server. Specifically, each user $U_{i}\in U$ encrypts his or her health data $m_{i,o}\in \left\{0,1,...,T\right\}$ at time point $t_{o}$ through the following three steps:

- Step 1: $U_{i}$ computes the hash value $\theta_{o}=H\left(t_{o}\right)$ at the time point $t_{o}$.
- Step 2: $U_{i}$ encrypts the message $m_{i,o}$ through calculating $C_{i,o}=g^{m_{i,o}}h^{\theta_{o}\cdot r_{i,o}}$, where $r_{i,o}\in Z_{n}^{+}$ is a random number. Furthermore, we set $h=g^{\alpha q}$ for some (unknown) α∈Z.
- Step 3: $U_{i}$ submits the ciphertext $C_{i,o}\in G$ to one of the working cloud server.

TA generates ciphertext of W: The weight vector directly reflects the importance of each message and the reliability of every user. Therefore, W should be encrypted, as well. Specifically, TA encrypts W at time point $t_{o}$ through the following three steps:

- Step 1: TA computes the hash value $\eta_{o}=H\left(t_{o}\right)$ at the time point $t_{o}$.
- Step 2: TA encrypts the *i*-th weight $w_{i,o}$ through calculating ${\tilde{w}}_{i,o}=g^{w_{i,o}}h^{\eta_{o}\cdot \rho_{i,o}}$, where $\rho_{i,o}\in Z_{n}^{+}$ is a random number. Furthermore, we set $h=g^{\alpha q}$ for some (unknown) α∈Z. Thus, the weight vector’s ciphertext is $\tilde{W}=\left({\tilde{w}}_{1,o},{\tilde{w}}_{2,o},...,{\tilde{w}}_{k,o}\right)$
- Step 3: TA submits the ciphertext $\tilde{W}\in G$ to one of the working cloud server.

Privacy-preserving computing of ${\mathrm{Sum}}_{w}$: One of the working cloud servers receives all of the users’ encrypted health data $\tilde{M}=\left(C_{1,o},C_{2,o},...,C_{k,o}\right)$ and $\tilde{W}=\left({\tilde{w}}_{1,o},{\tilde{w}}_{2,o},...,{\tilde{w}}_{k,o}\right)$. According to the privacy requirements, the cloud server needs to compute the encrypted weighted sum without knowing the plaintext of any specific message. By utilizing the homomorphic property of Boneh–Goh–Nissim, we can obtain the encrypted aggregation sum $Sum_{w}$ as follows.

As introduced in Section 3.1, anyone can multiply two encrypted messages once using the bilinear map. Let $g_{1}=e\left(g,g\right)$ and $h_{1}=e\left(g,h\right)$. Then, we have:

(11) $$\begin{array}{cc} e\left(C_{i,o},{\tilde{w}}_{i,o}\right)=e\left(g^{m_{i,o}}h^{\theta_{o}\cdot r_{i,o}},g^{w_{i,o}}h^{\eta_{o}\cdot \rho_{i,o}}\right) & =g_{1}^{w_{i,o}m_{i,o}}h_{1}^{m_{i,o}\eta_{o}\rho_{i,o}+w_{i,o}\theta_{o}r_{i,o}+\alpha q\theta_{o}r_{i,o}\eta_{o}\rho_{i,o}}\\ & =g_{1}^{m_{i,o}w_{i,o}}h_{1}^{{\bar{r}}_{i}}\;\;\;\in \;\;\;G_{1} \end{array}$$

where ${\bar{r}}_{i}=m_{i,o}\eta_{o}\rho_{i,o}+w_{i,o}\theta_{o}r_{i,o}+\alpha q\theta_{o}r_{i,o}\eta_{o}\rho_{i,o}$. We note that the cryptosystem is still additively homomorphic in $G_{1}$. Thus, we can compute $Sum_{w}$ as:

(12) $$Sum_{w}=\prod_{i=1}^{k}e\left(C_{i,o},{\tilde{w}}_{i,o}\right)=\prod_{i=1}^{k}g_{1}^{m_{i,o}w_{i,o}}h_{1}^{{\bar{r}}_{i}}=g_{1}^{\sum_{i=1}^{k}m_{i,o}w_{i,o}}h_{1}^{\sum_{i=1}^{k}{\bar{r}}_{i}}\;$$

Since the plaintext of weighted sum $\sum^{*}=W\cdot M=\sum_{i=1}^{k}m_{i,o}w_{i,o}$, we have:

(13) $$Sum_{w}=g_{1}^{\sum^{*}}h_{1}^{\sum_{i=1}^{k}{\bar{r}}_{i}}\;$$

Joint decryption of ${\mathrm{Sum}}_{w}$: Exactly the same as the basic scheme, TA randomly chooses l+1 cloud servers δ∈S as the working servers. As mentioned above, one cloud server calculates $Sum_{w}$ and sends it to the other *l* working cloud servers. Upon receiving $Sum_{w}$ at time point $t_{o}$, l+1 cloud servers decrypt $Sum_{w}$ together. Eventually, we have:

(14) $$\begin{array}{c} D_{o}=Sum_{w}^{p}={\left(g_{1}^{\sum^{*}}\right)}^{p}\cdot {\left(h_{1}^{\sum_{i=1}^{k}{\bar{r}}_{i}}\right)}^{p}={\left(g_{1}^{p}\right)}^{\sum^{*}}={\hat{g_{1}}}^{\sum^{*}} \end{array}$$

where $\hat{g_{1}}=g_{1}^{p}$.

Since $m_{i},w_{i}\in \left\{0,1,2,...,T\right\}$, we have $\sum^{*}=\sum_{i=1}^{k}m_{i,o}w_{i,o}\leq \left(k+1\right)T^{2}$. Nevertheless, *T* is the range of the message, and it is large enough; we let $m_{i,o}w_{i,o}\leq T$. Thus, the CS can obtain the weighted sum of users’ health data $\sum^{*}$, by computing the discrete logarithm of $D_{o}$ based on $\hat{g_{1}}$ in the expected time $O(\sqrt{(k+1)T})$. At last, the CS sends $\sum^{*}$ to TA.

Result release under differential privacy: Similar to the basic scheme, TA is responsible for computing the final result and releasing it under differential privacy. Once TA receives $\sum^{*}$, TA can get the weighted average value by simply calculating $M=\frac{\sum^{*}}{k}$. Generally, TA adds noise to *M* by leveraging the Laplace mechanism. Let Z be a random variable, and $Z\in Lap(\frac{T\cdot w_{max}}{\epsilon (\sum_{weight}-w_{max})})$. Here, Z is the Laplace noise, which will be added to *M*, and $w_{max}$ is the largest weight of users. Then, TA simply computes $\tilde{M}=M+Z$. Finally, $\tilde{M}$ is returned to the data analyst (HIs) as the final result. We can assert that the released result $\tilde{M}$ is *ϵ*-differentially private. The formal proof of differential privacy is given in Section 5.

#### 4.1.4. Privacy-Enhanced Average Aggregation Scheme

In the basic and weighted average aggregation schemes, one of the cloud servers is able to learn the sum of users’ data. Consequently, the cloud server may leak the sensitive statistical information to the unauthorized users. On addressing the problem, we design a privacy-enhanced average aggregation protocol.

First of all, let us recall the basic aggregation scheme (BAAS). Once all of the encrypted data are gathered by several cloud servers, one of the randomly chosen servers can access the decrypted summation of the dataset. In this scheme, all of the cloud servers are curious about the content of the data. Hence, the plaintext of the summation is under the risk of being disclosed. PAAS is designed to address the above problem.

In the initial phase, TA generates additional private keys for each user. Then, each user encrypts the message using private keys. The user generates two ciphertexts for one message and sends them to the cloud. The cloud then calculates two summations for one dataset. Since both summations are permutated by the additional private keys, the cloud cannot access the real summation of the dataset. TA recovers the original summation by solving an equation set. Actually, TA only needs to compute a polynomial.

Details of the scheme are shown as follows.

TA generates private keys for users: TA firstly generates additional private keys for mobile users. In order to strictly protect the privacy of the user, TA assigns each user a unique ID. A user’s ID could be a function of the serial number *i*. For instance, the *i*-th registered user’s ID can be set as $ID=i^{2}+i+1$ or some other functions. For simplicity, we set ID=i. Thus, each user’s ID just is the serial number *i*. Then, TA generates the *i*-th user’s private keys $X_{i}$ and $Y_{i}$ through computing $X_{i}=a_{1}i+b_{1},\;Y_{i}=a_{2}i+b_{2}$. Here, $a_{1},a_{2},b_{1},b_{2}$ are four random integers created by TA. Then, TA sends $\left(X_{i},Y_{i}\right)$ to the *i*-th user as its additional private keys.

User generates ciphertext: Each user $U_{i}\in U$ encrypts his or her health data $m_{i}$ at time point $t_{o}$ through the following three steps:

- Step 1: $U_{i}$ computes the hash value $\theta_{o}=H\left(t_{o}\right)$ at the time point $t_{o}$.
- Step 2: $U_{i}$ encrypts $m_{i,o}$ through calculating ${\tilde{m}}_{i,o}=g^{m_{i,o}}h^{\theta_{o}\cdot r_{i,o}}$, where $r_{i,o}\in Z_{n}^{+}$ is a random number. Furthermore, we set $h=g^{\alpha q}$ for some (unknown) α∈Z. Moreover, private keys $\left(X_{i},Y_{i}\right)$ are encrypted in the same way ${\tilde{X}}_{i}=g^{X_{i}}h^{\theta_{o}\cdot r_{i,o}},\;{\tilde{Y}}_{i}=g^{Y_{i}}h^{\theta_{o}\cdot r_{i,o}}$.
- Step 3: Let $g_{1}=e\left(g,g\right)$ and $h_{1}=e\left(g,h\right)$. $U_{i}$ creates the ciphertexts $\left(C_{i,o,1},C_{i,o,2}\right)$ of $m_{i,o}$ as follows.
  (15) $$\left\{\begin{array}{c} C_{i,o,1}=e\left({\tilde{m}}_{i,o},{\tilde{X}}_{i}\right)=e\left(g^{m_{i,o}}h^{\theta_{o}\cdot r_{i,o}},g^{X_{i}}h^{\theta_{o}\cdot r_{i,o}}\right)=g_{1}^{m_{i,o}X_{i}}\cdot h_{1}^{{\bar{r}}_{i,1}}\\ C_{i,o,2}=e\left({\tilde{m}}_{i,o},{\tilde{Y}}_{i}\right)=e\left(g^{m_{i,o}}h^{\theta_{o}\cdot r_{i,o}},g^{Y_{i}}h^{\theta_{o}\cdot r_{i,o}}\right)=g_{1}^{m_{i,o}Y_{i}}\cdot h_{1}^{{\bar{r}}_{i,2}} \end{array}\right.$$
  where ${\bar{r}}_{i,1}=m_{i,o}\theta_{o}r_{i,o}+X_{i}\theta_{o}r_{i,o}+\alpha q{\left(\theta_{o}r_{i,o}\right)}^{2}$ and ${\bar{r}}_{i,2}=m_{i,o}\theta_{o}r_{i,o}+Y_{i}\theta_{o}r_{i,o}+\alpha q{\left(\theta_{o}r_{i,o}\right)}^{2}$.
- Step 4: $U_{i}$ submits the ciphertexts $\left(C_{i,o,1},C_{i,o,2}\right)$ of $m_{i}$ to one working cloud server $S_{j}\in S$.

Privacy-preserving computing of ${\mathrm{Sum}}_{1}$ and ${\mathrm{Sum}}_{2}$: $S_{j}$ receives all of the users’ encrypted health data. Since each user submits two ciphertexts $\left(C_{i,o,1},C_{i,o,2}\right)$ of the message $m_{i}$, $S_{j}$ needs to compute the encrypted sum twice. By utilizing the homomorphic property of Boneh–Goh–Nissim, we can obtain the encrypted aggregation sum $Sum_{1}$ and $Sum_{2}$ as follows,

(16) $$\left\{\begin{array}{c} Sum_{1}=\prod_{i=1}^{k}C_{i,o,1}=\prod_{i=1}^{k}\left(g_{1}^{m_{i,o}X_{i}}\cdot h_{1}^{{\bar{r}}_{i,1}}\right)=g_{1}^{\sum_{i=1}^{k}m_{i,o}X_{i}}\cdot h_{1}^{\sum_{i=1}^{k}{\bar{r}}_{i,1}}\\ Sum_{2}=\prod_{i=1}^{k}C_{i,o,2}=\prod_{i=1}^{k}\left(g_{1}^{m_{i,o}Y_{i}}\cdot h_{1}^{{\bar{r}}_{i,2}}\right)=g_{1}^{\sum_{i=1}^{k}m_{i,o}Y_{i}}\cdot h_{1}^{\sum_{i=1}^{k}{\bar{r}}_{i,2}} \end{array}\right.$$

Joint decryption of ${\mathrm{Sum}}_{1}$ and ${\mathrm{Sum}}_{2}$: As mentioned above, the cloud sever $S_{j}$ calculates $Sum_{1}$ and $Sum_{2}$. Then, $S_{j}$ sends them to the other *l* working cloud servers. Upon receiving $Sum_{1}$ and $Sum_{2}$ at time point $t_{o}$, l+1 cloud servers decrypt $Sum_{1}$ and $Sum_{2}$ together. Finally, we have:

(17) $$\left\{\begin{array}{c} D_{1}=Sum_{1}^{p}={\left(g_{1}^{\sum_{i=1}^{k}m_{i,o}X_{i}}\right)}^{p}\cdot {\left(h_{1}^{\sum_{i=1}^{k}{\bar{r}}_{i,1}}\right)}^{p}={\left(g_{1}^{p}\right)}^{\sum_{i=1}^{k}m_{i,o}X_{i}}={\hat{g_{1}}}^{\sum_{i=1}^{k}m_{i,o}X_{i}}\\ D_{2}=Sum_{2}^{p}={\left(g_{1}^{\sum_{i=1}^{k}m_{i,o}Y_{i}}\right)}^{p}\cdot {\left(h_{1}^{\sum_{i=1}^{k}{\bar{r}}_{i,2}}\right)}^{p}={\left(g_{1}^{p}\right)}^{\sum_{i=1}^{k}m_{i,o}Y_{i}}={\hat{g_{1}}}^{\sum_{i=1}^{k}m_{i,o}Y_{i}} \end{array}\right.$$

Since $m_{i},X_{i},Y_{i}\in \left\{0,1,2...,T\right\}$, we have $\sum_{X}=\sum_{i=1}^{k}m_{i,o}X_{i}\leq \left(k+1\right)T^{2}$ and $\sum_{Y}=\sum_{i=1}^{k}m_{i,o}Y_{i}\leq \left(k+1\right)T^{2}$. Nevertheless, *T* is the range of the message, and it is large enough; we let $m_{i,o}X_{i}\leq T$ and $m_{i,o}Y_{i}\leq T$. Thus, the CS can obtain $\sum_{X}$ and $\sum_{Y}$ by computing the discrete logarithm of $D_{1}$ and $D_{2}$ based on $\hat{g_{1}}$ in expected time $O(\sqrt{(k+1)T})$. At last, the CS sends $\sum_{X}$ and $\sum_{Y}$ to TA.

Result release under differential privacy: TA is responsible for computing the final result and releasing it under differential privacy. Once TA receives $\sum_{X}$ and $\sum_{Y}$, TA can get the data sum $\sum_{i=1}^{k}m_{i,o}$ by solving the following equation set,

(18) $$\left\{\begin{array}{c} \sum_{X}=\sum_{i=1}^{k}m_{i,o}X_{i}\\ \sum_{Y}=\sum_{i=1}^{k}m_{i,o}Y_{i}\\ X_{i}=a_{1}i+b_{1}\\ Y_{i}=a_{2}i+b_{2} \end{array}\right.$$

Based on the above equation set, we can deduce that:

(19) $$\left\{\begin{array}{c} \sum_{X}=a_{1}\sum_{i=1}^{k}m_{i,o}\cdot i+b_{1}\sum_{i=1}^{k}m_{i,o}\\ \sum_{Y}=a_{2}\sum_{i=1}^{k}m_{i,o}\cdot i+b_{2}\sum_{i=1}^{k}m_{i,o} \end{array}\right.$$

Here, $a_{1},a_{2},b_{1},b_{2}$ are four integers generated by TA. Therefore, the solution of the equation set is $\sum_{i=1}^{k}m_{i,o}=\left(a_{2}\sum_{X}-a_{1}\sum_{Y}\right)/\left(a_{2}b_{1}-a_{1}b_{2}\right)$. Therefore, the average value of the dataset is $M\;=\;(\sum_{i=1}^{k}m_{i,o})/k$.

Similarly, TA adds noise to *M* by utilizing the Laplace mechanism. Let Z be a random variable, and $Z\in Lap(\frac{T}{\epsilon (k-1)})$. Z is the Laplace noise, which will be added to *M*. Then, TA simply calculates $\tilde{M}=M+Z$. Finally, M is returned to the data analyst (HIs) as the final result. Moreover, $\tilde{M}$ is *ϵ*-differentially private. Since the aggregation function of PAAS is exactly the same as BAAS, the differential privacy is guaranteed.

### 4.2. Non-Additive Aggregation of Health Data

In reality, non-additive aggregation results are widely applied in cloud-assisted WBAN systems; for instance, min/max, median, histogram, etc. In this section, we propose a scheme for these aggregation tasks. The same as the scheme proposed in [6], the comparison of the data is accomplished in the plaintext environment. In fact, if one wants to obtain the non-additive aggregation results of a encrypted dataset without decrypting it, order-preserving encryption techniques may be applied. Because the comparison is the basic operation, which cannot be finished without knowing the order information of the data, therefore the decryption of the specific message in the cloud servers is inevitable.

Different from [6], all of the decrypted messages $m_{i}\in \left\{m_{1},m_{2},...,m_{k}\right\}$ are gathered by TA. Thus, the distribution of the data is protected by TA, and cloud servers cannot get access to it. First, one user $U_{i}\in U$ encrypts its message $m_{i}$. Second, $U_{i}$ sends $Enc(m_{i})$ to the cloud server. Here, we use $Enc(m_{i})$ to represent the ciphertext of $m_{i}$. Third, l+1 working cloud servers are randomly chosen to decrypt the message. At last, the plaintext of each message $m_{i}$ is uploaded to TA. Thus, TA gathers all of the users’ messages $M=\{m_{1},m_{2},...,m_{k}\}$. In [6], the scheme stores all of the plaintext of the messages in one cloud server. If this cloud server is compromised, all of the users’ personal health information may be leaked. For the privacy consideration, we let TA store the plaintext of the whole dataset.

Once TA has gathered all of the users’ messages M, it sorts M’s elements. Then, TA uses an array A[k+1] to store the ordered messages. For instance, the minimum message is A[1], and the maximum is A[k]. Note that the first element of array A is empty. Therefore, we have A[1]<A[2]<...<A[k].

Based on the ordered array A, we can easily deduce that the median message is $med=A[\frac{k+1}{2}]$, if *k* is odd. When *k* is even, $med=(A\left[\frac{k}{2}\right]+A\left[\frac{k}{2}+1\right])/2$. As mentioned above, the minimum and maximum messages are min=A[1], max=A[k]. In [6], noises are added to the result of min/max and median aggregations. However, the scale of the noises is too large, because the sensitivities of min/max and median aggregations are max{M}=T. Since healthcare institutions (HIs) provide health services based on these data, the min/max and median values of users’ health data are required to be as accurate as possible. Thus, TA releases min/max and med directly to the HIs. In the following paragraphs, we will show how to accomplish histogram aggregation and release it with differential privacy guaranty.

#### 4.2.1. Hierarchical Method for Histogram

**Figure 2:**
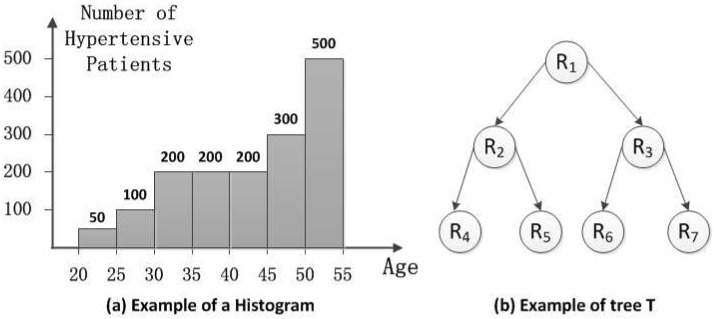
(**a**) Example of a histogram. (**b**) Example of query tree T.

In this part, we provide the histogram aggregation protocol. The histogram is widely used to reflect the distribution of dataset in statistics. Specifically, each bin of a histogram illustrates the frequency of the values falling into it. As shown in Figure 2a, the first bin’s value is 50. Each bin’s value is the count of the hypertensive patients of a certain age group. Since TA has the ordered messages A[1],A[2],...,A[k], it can easily obtain the values of the bins in the histogram. Then, TA answers the query submitted by the healthcare institutions based on the histogram. Note that the original histogram can directly reflect the distribution of the dataset. In order to protect the sensitive information, we add noises to the result of the query. Besides, we leverage the post-processing technique [12] to boost the accuracy. Details of query sequence generation, the differential privacy mechanism and post-processing are shown in the following paragraphs.

Query sequence generation: A query sequence is proposed by the HIs, which not only asks for unit-length intervals (e.g., single data bin), but also asks for larger intervals (e.g., age from 20–45 in Figure 2a). In this strategy, the larger intervals are a linear combination of the few counts of sub-intervals. The query sequence consists of a batch of hierarchical intervals denoted as a vector H.

Moreover, the intervals can be arranged into a tree *T*, where the unit-length intervals are the leaves. Each node v∈T in the tree corresponds to an interval with *s* children. The node *v*’s children are equally-sized sub-intervals. We use H to denote the case of a query tree with *n* leaves. If *t* denotes the height of the tree and *s* is the branch factor, then $\left\lceil t=log_{s}n+1\right\rceil$. The sequence is ordered by a breadth-first traversal of the tree. Here, we give an example of tree *T* associated with query sequence H for s=2.

As shown in Figure 2b, the query sequence is $H=\{R_{1},R_{2}...,R_{7}\}$. Each query is arranged into the tree *T* whose value is the sum of its children. For instance, the root ($R_{1}$) is the whole range of the histogram equal to $R_{2}+R_{3}$.

Once TA receives the query sequence H, it computes each query from the leaf to the root of tree *T*. We use H(A) to represent the result of query on message array A.

Add noises to the query sequence: To answer H under differential privacy, the first step is to analyze the sensitivity of H. When users alter one record, the count changes within ±1 through the same level. As the level of the tree is *t*, the output of H changes ±t at most. Thus, ΔH=t. By the aforementioned inference and basic Laplace mechanism, the following algorithm satisfies *ϵ*-differential privacy:

(20) $$\tilde{H}\left(A\right)=H\left(A\right)+{\left[Lap\left(t/\epsilon \right)\right]}^{m}\;$$

where *m* is the length of sequence H.

Problem of consistency: The property of consistency will be broken after adding noises to the query results. Because the noises added are randomly produced, there may be a noisy count that does not equal the sum of its sub-intervals. Therefore, $\tilde{H}$ cannot be the final output.

Post-processing of query sequence: This step is aimed to derive a consistent and more accurate query result denoted as $\bar{H}$. We adopt the method proposed by [12]. As summarized in [18], this technique can be dissected into two steps: weighted averaging and mean consistency.

Weighted averaging: First, we need to ensure a fact depicted as the following sentences:

Given two random variables *X* and *Y*, consider Z=αX+(1−α)Y. When $\alpha =\frac{Var(Y)}{Var(X)+Var(Y)}$, the variance of *Z* gets the minimum and minimal variance, which is $\frac{Var(X)Var(Y)}{Var(X)+Var(Y)}$.

Let $\tilde{H}$ be the noisy value of node v∈T at level *t* of tree *T*. To estimate the node’s noisy value, we use the weighted average of its original noisy value and the sum of its children’s count. Let z[v] be the transformed count and succ(v) be the set of *v*’s children.

(21) $$z\left[v\right]=\left\{\begin{array}{c} \tilde{H}\left[v\right],\;if\;v\;is\;a\;leaf\;node\\ \frac{s^{t}-s^{t-1}}{s^{t}-1}\tilde{H}\left[v\right]+\frac{s^{t-1}-1}{s^{t}-1}\sum_{u\in succ(v)}z\left[u\right],o.w. \end{array}\right.$$

Mean consistency: The mean consistency step aims at ensuring that for each node, its children values sum up to be the same as the parent. Let *u* be the parent of *v*.

(22) $$\bar{H}\left[v\right]=\left\{\begin{array}{c} z[v],\;if\;v\;is\;the\;root\\ z\left[v\right]+\frac{1}{s}\left(\bar{H}\left[u\right]-\sum_{w\in succ(u)}z\left[w\right]\right),o.w. \end{array}\right.$$

After post-processing, each node’s value is a weighted sum of the original noisy values of all nodes in the tree. At last, TA returns the final result $\bar{H}$ to HIs. The formal analysis of differential privacy is given in Section 5.

## 5. Security and Privacy Analysis

In this section, we will discuss the security and privacy issues involved in PMHA-DP. The adversary model, security and privacy requirements are given in Section 3. Clearly, our mission is to preserve users’ private health data from the adversary Adv and to satisfy all of the security requirements. As mentioned in Section 4, each aggregation scheme (includes BAAS, WAAS, PAAS, HMH) is under a differential privacy guarantee. Therefore, we will show the privacy proof of each scheme in this section. Details are shown as follows.

- The users’ privacy is preserved, even if the communication flows are intercepted by Adv. Specifically, Adv may eavesdrop on the communication flows from users to the cloud servers. However, the mobile users in WBANs are dynamic, and the number of users is large; it is impractical for Adv to do so. Even if the data are captured by Adv at time point $t_{o}$, $U_{i}$’s message $m_{i,o}$ is encrypted as $g^{m_{i,o}}\cdot h^{\theta_{o}r_{i,o}}$. Thus, Adv cannot decrypt the ciphertext and obtain the message $m_{i,o}$ without private key *p*. Therefore, we can assert that Adv cannot reveal the private data, even if the communication flows are intercepted.
- Adv cannot reveal the uncompromised users’ private health data. Since the amount of mobile users is quite large in the cloud-assisted WBANs system, Adv would be unlikely to breach users’ privacy through compromising some of the users. We assume that Adv may try to disclose the uncompromised users’ private health data by utilizing the private information and private keys of the compromised users. Namely, Adv is able to obtain some of the users’ private keys and their personal data. However, the privacy of uncompromised users is well guaranteed, and the reasons are listed as follows. First, each mobile user’s private key is generated independently; one can deduce nothing about another user from one user’s private key. Second, the sum of all users’ private keys is transparent to Adv. Even if Adv learns k−1 users’ private keys, it still cannot reveal the last user’s private key and health data.
- Adv cannot obtain users’ private health data and the aggregated data, even if *l* CSs are compromised. In the system initialization phase, TA distributes the private keys G(j),j=1,2,...,l to each CS and l≥3. In this system, the users’ privacy can be protected when no more than l=⌈n/2⌉−1 CSs are paralyzed or compromised. According to the “all or nothing” property of secret sharing [17], at least l+1 CSs are needed to acquire private key *p*. Therefore, even if Adv possesses *l* private keys, it still cannot recover *p*. Similarly, Adv only has *l* decryption shares of CSs at most, which are insufficient to recover the aggregated data either. Therefore, Adv cannot expose the aggregated data.
- In the privacy-enhanced scheme, Adv cannot acquire the aggregated data, even if all of the l+1 CSs are compromised. First, TA utilizes each user’s ID to generate $U_{i}$’s additional private key $\left(X_{i},Y_{i}\right)$ and distributes them through a secure channel. Then, $U_{i}$ encrypts its message $m_{i}$ twice by using *p* and $\left(X_{i},Y_{i}\right)$. Then, the l+1 working CSs calculate two encrypted sums of all of the data. Moreover, l+1 working CSs cannot recover the real sum without knowing each user’s additional private key $\left(X_{i},Y_{i}\right)$ and four randomly generated numbers $a_{1},a_{2},b_{1},b_{2}$. Even if all of these secure parameters and two encrypted sums are leaked to Adv, it still does not know how to build an equation group and compute the real sum. Consequently, the aggregated data are well protected even if all of the l+1 CSs are compromised by Adv.
- Adv cannot deduce any individual user’s health data by launching differential attacks on TA. TA is the only entity that can release statistical information to HIs. TA adds Laplace noises to the original aggregated data before release. Therefore, the differential privacy mechanism is only applied on TA. Due to the property of the Laplace mechanism, TA is able to resist differential attacks in each proposed aggregation scheme. The proof of differential privacy is given below.

Here, we give a formal differential privacy proof of each aggregation scheme. First, the proof of BAAS is shown as follows:

**Proof** **(Proof of Differential Privacy).** BAAS directly utilizes the Laplace mechanism [13], which is introduced in Definition 3. Thus, the critical step of privacy proof is calculating the sensitivity of the aggregation function. Here, the output of the function is the average of a dataset. Therefore, we rewrite the function as $f\left(D\right)=\frac{\sum_{i=1}^{k}m_{i}}{k}$. Here, $m_{i}$ is the *i*-th user’s record of the dataset *D*, and *k* is the total number of the users’ data. Let $D^{\prime }\in nbrs\left(D\right)$ be the neighboring dataset differing from D in at most one record. Therefore, if we want to calculate $\Delta f=\max_{D,D^{\prime }}{\left|f\left(D\right)-f\left(D^{\prime }\right)\right|}_{1}$, we need to analyze the problem from three conditions.

**Condition** **1.** *$D^{\prime }$ has one more record m than D. Consider two extreme situations, m=0 and m=max, where max denotes the largest value of the user’s record. If m=0, $|f\left(D\right)-f\left(D^{\prime }\right)|=f\left(D\right)-\frac{kf(D)}{k+1}=\frac{f(D)}{k+1}$. If m=max, $|f\left(D\right)-f\left(D^{\prime }\right)|=\frac{kf(D)+max}{k+1}-f\left(D\right)=\frac{max-f(D)}{k+1}$. Therefore, we have:*

(23) $$\Delta f_{1}=\max_{0\leq f(D)\leq max}\left\{\frac{f(D)}{k+1},\frac{max-f(D)}{k+1}\right\}=\frac{max}{k+1}\;$$

**Condition** **2.** *$D^{\prime }$ has one less record m than D. If m=0, $|f\left(D\right)-f\left(D^{\prime }\right)|=\frac{kf(D)}{k-1}-f\left(D\right)=\frac{f(D)}{k-1}$. If m=max, $|f\left(D\right)-f\left(D^{\prime }\right)|=f\left(D\right)-\frac{kf(D)-max}{k-1}=\frac{max-f(D)}{k-1}$. Thus, we have:*

(24) $$\Delta f_{2}=\max_{0\leq f(D)\leq max}\left\{\frac{f(D)}{k-1},\frac{max-f(D)}{k-1}\right\}=\frac{max}{k-1}$$

**Condition** **3.** D and $D^{\prime }$ have the same number of records, but m∈D and $m^{\prime }\in D^{\prime }$ are the only different records between them. If m=max and $m^{\prime }=0$, $|f\left(D\right)-f\left(D^{\prime }\right)|=f\left(D\right)-\frac{kf(D)-max}{k}=\frac{max}{k}$. If, m=0 and $m^{\prime }=max$, $|f\left(D\right)-f\left(D^{\prime }\right)|=\frac{kf(D)+max}{k}-f\left(D\right)=\frac{max}{k}$. Then, we have $\Delta f_{3}=\frac{max}{k}$.

*Based on the above analysis, we can deduce that:*

(25) $$\Delta f=\max_{D,D^{\prime }}{\left|f\left(D\right)-f\left(D^{\prime }\right)\right|}_{1}=\max\left\{\Delta f_{1},\Delta f_{2},\Delta f_{3}\right\}=\frac{max}{k-1}$$

Since $m_{i}\in \left\{0,1,2,...,T\right\}$, max=T. Thus, the sensitivity of function *f* is $\Delta f=\frac{T}{k-1}$. According to Definition 3, the algorithm $\tilde{M}=M+Z\;\;s.t.\;\;Z∼Lap\left(\frac{T}{\epsilon (k-1)}\right)$ is *ϵ*-differentially private.  ☐

The above analysis indicates that quantifying the sensitivity of the aggregation function is critical. The sensitivity analysis of the weighted average aggregation function (i.e., WAAS) is almost the same as BAAS. Thus, we omit it. Moreover, since the aggregation function of PAAS is exactly the same as BAAS, the differential privacy is guaranteed.

The formal analysis of the differential privacy of HMH is shown as follows:

**Proof** **of Differential Privacy.** The sensitivity of query sequence H is well analyzed in Section 4.2. We formalize it as ΔH=t. According to the Laplace mechanism [13], when the added random variable satisfies Lap(ΔH/ϵ), the algorithm is *ϵ*-differential privacy. Note that the post-processing does not consume the privacy budget.  ☐

## 6. Performance Evaluation

In this section, we evaluate the performance of PMHA-DP in terms of functionality, computation and communication overhead. Besides, the error analysis is also provided. We will compare our additive and non-additive aggregation schemes with ${\mathrm{MHDA}}^{+}$[6] and ${\mathrm{MHDA}}^{⊕}$ [6].

Here, we will give a detailed discussion on the dataset. As we know, once the differential privacy mechanism is chosen, the noise scale is also calibrated. In this paper, we utilize the Laplace mechanism as the building block of the scheme. Hence, the sensitivity of the aggregation function is the only parameter that can directly affect the noise scale (i.e., the accuracy of the result). In reality, there are many different health data, such as the blood pressure, body temperature, heart rate, and so on. Different data types lead to different data ranges and typical sizes. For instance, usually, body temperature is within the range of 35–45 degrees Celsius. For a common person, the mean value of the body temperature is 36.9 Celsius. However, for another kind of health data, such as blood pressure, the data range must be totally different from the body temperature. The maximum of blood pressure should be less than 180 mmHg. In this paper, the maximum of the message should be less than *T*. Hence, our scheme can be applied for different data types as long as the max value is less than *T*. In this paper, we only consider the size of the largest message to evaluate the sensitivity and the error of the result. Therefore, the error of each protocol is irrelevant to the type of data. Specifically, we vary the size of the message *w* ($T=2^{w}-1$) from {12, 13, 14, 15, 16, 17, 18, 19, 20, 21} (*w* is the length of a message). Once the maximum value and the message amount are both established, we can calculate the mathematical expectation of the square difference of the Laplace mechanism.

### 6.1. Functionality

In our additive aggregation scheme, we propose a basic scheme that is similar to ${\mathrm{MHDA}}^{+}$. Both our scheme and ${\mathrm{MHDA}}^{+}$ provide an average aggregation protocol. In reality, the weighted average is also widely used. However, to the best knowledge of the authors, their are few reported works in the open literature providing a privacy-preserving weighted average aggregation scheme. We design a weighted average aggregation protocol and provide the final result under differential privacy. Moreover, as the working cloud servers in ${\mathrm{MHDA}}^{+}$ are able to learn the real value of the aggregated data, some sensitive information may be leaked. On addressing the above problem, we design a privacy-enhanced average aggregation scheme, which protects the real sum of the dataset from all of the cloud servers. Besides, the released result also satisfies differential privacy. The comparison of the functionality between PMHA-DP and ${\mathrm{MHDA}}^{+}$ is shown in Table 1.

The same as ${\mathrm{MHDA}}^{⊕}$, our non-additive aggregation scheme provides max/min, median and histogram aggregation protocols. As analyzed in Section 4.2, the scale of noise is too large for max/min and median aggregations. Thus, we release the final results without adding noises. Histogram aggregation is well discussed in our paper. We first apply the hierarchical method to answer the query submitted by HIs. Then, for privacy consideration, we add Laplace noises to the result. Furthermore, we leverage the post-processing technique to boost the accuracy of noisy answers. Thus, our histogram aggregation scheme is more practical. The comparison of the functionality between PMHA-DP and ${\mathrm{MHDA}}^{⊕}$ is shown in Table 2.

### 6.2. Computational Overhead

For additive aggregation schemes, the computation overhead is clear. Because, the process of the encryption and decryption of the whole aggregation system can be divided into some basic calculations, in this paper, we mainly consider four different kinds of calculations. As shown in Table 3, we use some symbols to denote the time of each operation.

In our system, there are three entities that share the total computational overhead: MUs (mobile users), TA and CSs. Consequently, we analyze the computational overhead of ${\mathrm{MHDA}}^{+}$ and three different additive aggregation protocols of PMHA-DP in the above three aspects. Details are shown as follows.

In this paper, the TA has to bear some computational tasks. We have tried our best to reduce the computational burden of TA. As we know, the encrypted data are outsourced to the public cloud. Most aggregation operations are finished by the cloud. However, the cloud servers are honest-but-curious, which may learn the content of the data. Therefore, some sensitive information of the dataset should not be disclosed to the cloud servers. TA is assigned to add noises to the original query result, such as the summation of the data. Due to the property of the Laplace mechanism, the noise scale is proportional to the sensitivity of the aggregation function. Once the privacy budget is set, it is easy to obtain the sensitivity. The sensitivity of an aggregation function can directly reflect the distribution of a dataset. For instance, if the sensitivity of a function is about 37, we can deduce that the dataset is the body temperature. Furthermore, the TA only needs to add noises to the final result, and it is irrelevant regarding the number of users. The time complexity of adding noises is O(1).

For WAAS, TA needs to store the weights of the users and encrypt them before sending them to the cloud servers. Each user’s weight is personal information, and it should be protected from the public cloud. When the system is running for the first time, TA needs to encrypt all of the users’s weights. After the first time, TA only needs to do some partial modifications, which can significantly reduce the computational burden.

For PAAS, the additional calculation of TA is computing the summation of the data. TA can obtain the sum by calculating a simple polynomial. The time complexity is O(1).

Computation overhead of BAAS: Each MU encrypts the health data with one modular multiplication and two modular exponential operations. Therefore, each MU’s total computational overhead is $2T_{exp}+T_{mul}$. One of the working CSs gathers all of the MUs’ messages by taking k−1 modular multiplication, that is $\left(k-1\right)T_{mul}$. Then, all of the l+1 working CSs calculate the decryption shares with l+1 modular exponential operations. Next, one randomly chosen CS gathers the decryption, shares and decrypts it using Pollard’s lambda method, which takes $lT_{mul}+T_{pol}$. Therefore, the total computational overhead of working CSs is $\left(l+1\right)T_{exp}+\left(k+l-1\right)T_{mul}+T_{pol}$. TA’s computation burden is light and negligible in the basic scheme.

Computation overhead of WAAS: Each MU encrypts the private data with time $2T_{exp}+T_{mul}$. TA encrypts each MU’s weight with time $2T_{exp}+T_{mul}$, and there are *k* users. Therefore, the total time of TA is $2kT_{exp}+kT_{mul}$. Then, all of the working CSs calculate the encrypted weighted sum of the dataset with *k* bilinear map operations and k−1 modular multiplication, that is $kT_{bim}+\left(k-1\right)T_{mul}$. At last, l+1 CSs jointly decrypt the aggregated data with the same time of the basic scheme: $\left(l+1\right)T_{exp}+lT_{mul}+T_{pol}$. Therefore, the total computation overhead of CSs is $\left(l+1\right)T_{exp}+\left(k+l-1\right)T_{mul}+kT_{bim}+T_{pol}$.

Computation overhead of PAAS: Each user encrypts the message $m_{i,o}$ with time $2T_{exp}+T_{mul}$. Then, the user preprocesses the additional private keys $\left(X_{i},Y_{i}\right)$ with time $4T_{exp}+2T_{mul}$. Finally, the user generates the ciphertext of $m_{i,o}$ with two bilinear map operations. Thus, the total time of each user is $6T_{exp}+3T_{mul}+2T_{bim}$. In the privacy-enhanced scheme, working CSs need to calculate the sum of all users’ messages twice and jointly decrypt them. Therefore, the total computational overhead of CSs is two times that of the basic scheme, that is $2\left(l+1\right)T_{exp}+2\left(k+l-1\right)T_{mul}+2T_{pol}$. TA’s computation burden is negligible and is irrelevant to the scale of the dataset.

Computation overhead of ${\mathrm{MHDA}}^{+}$: In ${\mathrm{MHDA}}^{+}$[6], each individual MU encrypts the health data with two modular exponential operations and one modular multiplication. Therefore, the total overhead of an MU is $2T_{exp}+T_{mul}$. The aggregator SP’soverhead is $\left(k-1\right)T_{mul}$. The working CSs jointly decrypt the aggregated data with time $\left(l+1\right)T_{exp}+lT_{mul}+T_{pol}$.

The comparison of the computational overhead is shown in Table 4.

**Figure 3:**
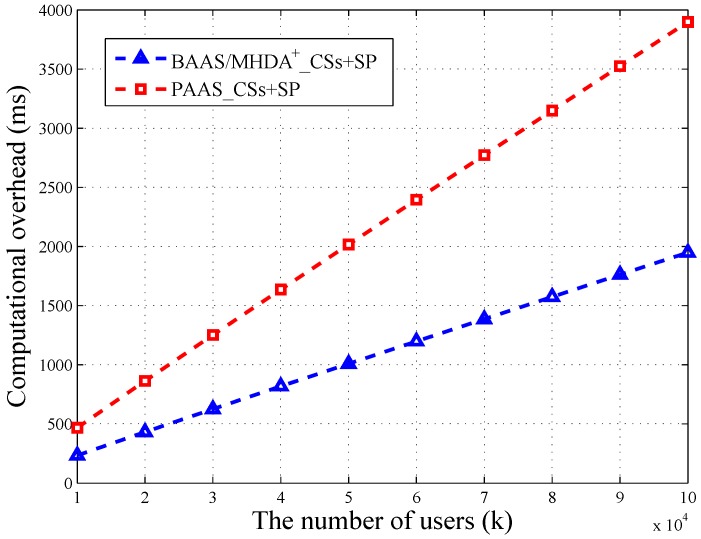
The computational overhead of BAAS, PAAS and ${\mathrm{MHDA}}^{+}$ [6].

**Figure 4:**
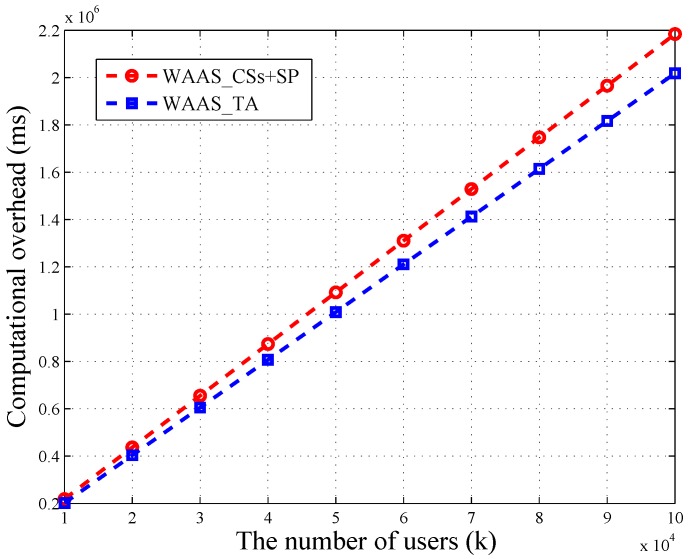
The computational overhead of TA and CSs + SP in WAAS.

We utilize OpenSSL Library [19] to conduct our experiment on a 2.65-GHz processor, 4 GB memory, computing machine. We let the security parameter τ=512 and set message’s bit length as w=13. Besides, we let the user number range from 10,000–100,000. One CS can provide service for 2500 users at most. The result of the experiment showed that the bilinear map operation and Pollard’s lambda method are quit time consuming. Specifically, we have the results $T_{exp}=10.082$ ms, $T_{mul}=0.016$ ms and $T_{bim}=21.823$ ms. Besides, $T_{pol}$ is directly proportional to $\sqrt{k(2^{w}-1)}$. When k=10,000, $T_{pol}=42.875$ ms. Based on these results, we depict the variation of computational overheads in terms of *k* in Figure 3 and Figure 4.

As shown in Figure 3, the computational overhead of CSs + SP in the basic scheme (BAAS) is exactly the same as ${\mathrm{MHDA}}^{+}$. Therefore, we use the same line to depict the variation. We can find that the overhead of the privacy-enhanced scheme (PAAS) is two times that of BAAS and ${\mathrm{MHDA}}^{+}$. When the user number is 100,000, PAAS’s computational overhead is 3898.80 ms, nearly 4 s. Besides, ${\mathrm{MHDA}}^{+}$’s overhead is 1949.40 ms, nearly 2 s. Therefore, the privacy-enhanced scheme takes up an extra 2 s of calculating time, which is acceptable. Even in the big data environment, suppose the user number reaches 10 million; the calculating time of PAAS is 363,159.64 ms, nearly six minutes. Consider that the high-performance servers in the cloud and part of the computation burden can be uniformly distributed; it is possible to reduce the running time into the acceptable range. Thus, PAAS protects the aggregated data at a low price.

As depicted in Figure 4, the time consumption of TA and CSs + SP in WAAS increases linearly with the number of users. Besides, the loads of TA and CSs are balanced. When the amount of users reaches 100,000, the computational overhead of TA is 2018 s, which is close to CSs’ 2184 s. In WAAS, CSs need to do *k* bilinear map operations, and TA is required to take 2k modular exponential operations. These two calculations are time consuming, but both of them can be completed in parallel. Thus, it is possible to reduce the running time.

### 6.3. Communication Overhead

First, we compare the communication overhead of the proposed additive aggregation schemes with ${\mathrm{MHDA}}^{+}$. As we know, the length of the ciphertext directly reflects the communication overhead. Therefore, the communication cost of each entity (TA MUs CSs) in BAAS is the same as ${\mathrm{MHDA}}^{+}$. In WAAS, the additional communication burden is carried by TA. TA needs to send all of the encrypted weights of MUs to CSs. Actually, in a system, one user’s weight cannot be set by himself or herself. Thus, some control centers like TA are bound to manage all of the users’ weight, and the communication cost is inevitable. In PAAS, each user needs to generate two ciphertexts for one message. Thus, the communication cost of MU is two times that of ${\mathrm{MHDA}}^{+}$.

For the proposed non-additive aggregation schemes (NAS) and ${\mathrm{MHDA}}^{⊕}$, the communication overhead can be considered in the communication of each MU. In NAS, MU reports its own encrypt message to CSs. However, MU in ${\mathrm{MHDA}}^{⊕}$ submits the ciphertext to SP instead. The length of MU’s ciphertext in ${\mathrm{MHDA}}^{⊕}$ is a linear function of the size of the plaintext *w*. That is 2τ(w+1), where *τ* is the security parameter. However, the size of MU’s ciphertext in NAS is a constant 2τ.

**Figure 5:**
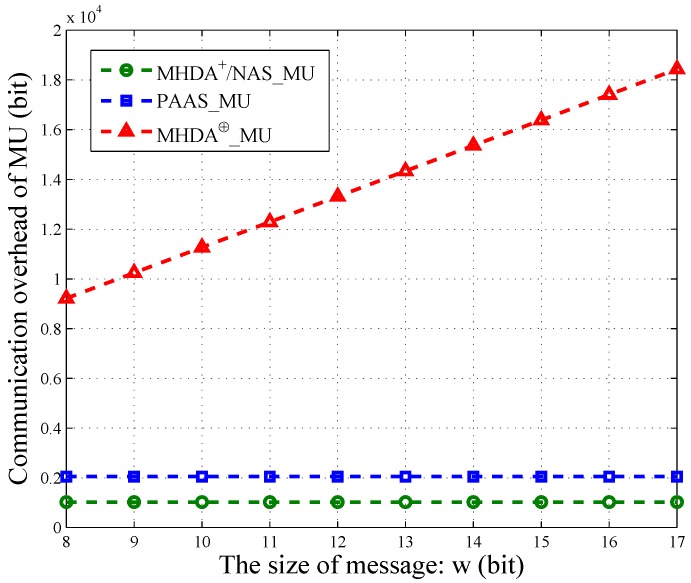
The communication overhead of MU in ${\mathrm{MHDA}}^{+}$ [6], ${\mathrm{MHDA}}^{⊕}$ [6], PAAS and NAS.

Actually, each MU’s packet also contains some other information, such as time stamp, user ID, and so on. However, the message occupies the most space of one packet. Therefore, we only consider the message’s communication overhead. As shown in Figure 5, we vary *w* from {8,9,10,11,12,13,14,15,16,17} and illustrate the communication cost of PAAS, NAS, ${\mathrm{MHDA}}^{+}$ and ${\mathrm{MHDA}}^{⊕}$ in terms of *w*. The size of MU’s encrypted data in NAS is the same as ${\mathrm{MHDA}}^{+}$. Thus, we use the same line to depict them. Similarly, the communication overhead of MU in PAAS is also a constant and is irrelevant to *w*. However, each MU in PAAS needs to generate two ciphertext for one message. Therefore, PAAS has twice the communication overhead of NAS and ${\mathrm{MHDA}}^{+}$, that is 4τ. For ${\mathrm{MHDA}}^{⊕}$, the overhead of MU increases linearly with the growing of *w*. Namely, the longer the message, the larger the communication overhead. Thus, NAS and PAAS are more practical when the message’s size is large.

### 6.4. Error Analysis

In our additive schemes, we directly add noise Z to the result *M*. Then, we obtain the noisy answer $\tilde{M}=M+Z$, where $Z∼Lap(\frac{\Delta f}{\epsilon })$. In this section, we use Δf to represent any aggregation function’s sensitivity. Thus, the mathematical expectation of the square difference of the Laplace mechanism is $error\left(\tilde{M}\right)=E{\left(\tilde{M}-M\right)}^{2}$, which simplifies to: $error\left(\tilde{M}\right)=E{\left(M+Z-M\right)}^{2}=E\left(Z^{2}\right)=Var\left(Lap\left(\frac{\Delta f}{\epsilon }\right)\right)$. Since $Var\left(Lap\left(\frac{\Delta f}{\epsilon }\right)\right)=2\frac{\Delta f^{2}}{\epsilon^{2}}$, we have $error\left(\tilde{M}\right)=2\frac{\Delta f^{2}}{\epsilon^{2}}$. As discussed in Section 4.1, the aggregation functions of BAAS and PAAS are the same, and the sensitivity of both functions is $\Delta f=\frac{T}{k-1}$. In WAAS, $\Delta f=\frac{T\cdot w_{max}}{\sum_{weight}-w_{max}}$. Thus, the errors of BAAS and PAAS are $error\left(BAAS\right)=error\left(PAAS\right)=\frac{2T^{2}}{\epsilon^{2}{\left(k-1\right)}^{2}}$. We also have $error\left(WAAS\right)=\frac{2T^{2}w_{max}^{2}}{\epsilon^{2}{\left(\sum_{weight}-w_{max}\right)}^{2}}$.

In our non-additive scheme, we choose not to add noise to the result of max/min and median aggregations; because the sensitivity of the function will be too large and is irrelevant to *k*, that is Δf=T. However, in ${\mathrm{MHDA}}^{⊕}$, the authors choose to add noises with a large scale. Therefore, we can deduce that the error of max/min and median aggregation in ${\mathrm{MHDA}}^{⊕}$ is $\frac{2T^{2}}{\epsilon^{2}}$. In the reality, the number of the users (*k*) is much larger than 10,000 or even more. Therefore, the error of max/min and median in ${\mathrm{MHDA}}^{⊕}$ should be larger than $10^{8}\;error\left(BAAS\right)$, which is unacceptable. For our histogram aggregation scheme HMH, the sensitivity is Δf=t, where *t* is the level of the query tree *T*. Thus, we have $error\left(HMH\right)=\frac{2mt^{2}}{\epsilon^{2}}$, where *m* is the length of the query vector H.

In ${\mathrm{MHDA}}^{+}$, the authors directly add noise to the sum of the health data. Therefore, the sensitivity is *T*. However, in ${\mathrm{MHDA}}^{+}$, the noisy sum is divided by *k* to acquire the noisy average. Therefore, we can deduce that the sensitivity of the mean value function in ${\mathrm{MHDA}}^{+}$ is $\Delta f=\frac{T}{k}$. Note that the noises in ${\mathrm{MHDA}}^{+}$ are sampled from the geometric distribution Geom(α). Its probability density function is:

(26) $$Pr\left(X=x\right)=\frac{1-\alpha }{1+\alpha }\alpha^{\left|x\right|}\;$$

where $\alpha =e^{-\frac{\epsilon }{\Delta f}}$ and 0<α<1. Let *X* be the noise sampled from Geom(α). Then, the error of ${\mathrm{MHDA}}^{+}$ is $error\left(MHDA^{+}\right)=E\left(X^{2}\right)$, which is calculated as follows.

(27) $$\begin{array}{cc} E(X^{2}) & =\sum_{x=-\infty }^{+\infty }x^{2}\frac{1-\alpha }{1+\alpha }\alpha^{\left|x\right|}=2\sum_{x=1}^{+\infty }x^{2}\frac{1-\alpha }{1+\alpha }\alpha^{x}=\frac{2}{1+\alpha }\left(\sum_{x=1}^{+\infty }x^{2}\alpha^{x}-\sum_{x=1}^{+\infty }x^{2}\alpha^{x+1}\right)\\ & =\frac{2}{1+\alpha }\left(\sum_{x=1}^{+\infty }\left(2x-1\right)\alpha^{x}\right)=\frac{2}{1+\alpha }\left(\sum_{x=1}^{+\infty }2x\alpha^{x}-\sum_{x=1}^{+\infty }\alpha^{x}\right) \end{array}$$

Let $u=\sum_{x=1}^{+\infty }2x\alpha^{x}$, and $v=\sum_{x=1}^{+\infty }\alpha^{x}=\frac{\alpha }{1-\alpha }$. We can deduce $u=2\alpha \left(\frac{dv}{d\alpha }\right)=2\alpha {\left(\frac{\alpha }{1-\alpha }\right)}^{\prime }=\frac{2\alpha }{{\left(1-\alpha \right)}^{2}}$. Then, we have $E\left(X^{2}\right)=\frac{2}{1+\alpha }\left[\frac{2\alpha }{{\left(1-\alpha \right)}^{2}}-\frac{\alpha }{1-\alpha }\right]=\frac{2\alpha }{{\left(1-\alpha \right)}^{2}}$. Since $\alpha =e^{-\frac{\epsilon }{\Delta f}}$ and $\Delta f=\frac{T}{k}$, the final result can be calculated as:

(28) $$error\left(MHDA^{+}\right)=E\left(X^{2}\right)=\frac{2e^{\frac{k\epsilon }{T}}}{e^{2\frac{k\epsilon }{T}}-2e^{\frac{k\epsilon }{T}}+1}$$

The errors of PMHA-DP and ${\mathrm{MHDA}}^{+}$ [6] are clearly listed in Table 5.

In Table 5, $w_{max}$ is the largest weight of all of the mobile users. Therefore, $w_{max}$ must be larger than the average of all of the users’ weights. Then, $\left(k-1\right)w_{max}\geq \sum_{weight}-w_{max}$, which can be transformed into $\frac{w_{max}}{\sum_{weight}-w_{max}}\geq \frac{1}{k-1}$. Therefore, the error of WAAS is greater than or equal to that of PAAS and BAAS. The noise of ${\mathrm{MHDA}}^{+}$ is sampled from the symmetric geometric distribution, which is a discrete approximation of the Laplace distribution. Consequently, the errors of ${\mathrm{MHDA}}^{+}$, BAAS and PAAS can be considered equivalent approximate. Let ϵ=0.1. We vary *k* from {10,000, 20,000, 30,000, 40,000, 50,000, 60,000, 70,000, 80,000, 90,000, 100,000} and vary *w* ($T=2^{w}-1$) from {12,13,14,15,16,17,18,19,20,21} to calculate the error of ${\mathrm{MHDA}}^{+}$ and BAAS/PAAS. All of the error values of ${\mathrm{MHDA}}^{+}$ and BAAS/PAAS are listed in Table 6. Apparently, the difference between error(BAAS/PAAS) and $error(MHDA^{+})$ is negligible.

Here, we give a detailed discussion on the dataset. As mentioned above, the sensitivity of the average aggregation function and the number of the messages are the only parameters that can directly affect the noise scale. In reality, there are many different health data, such as blood pressure, body temperature, heart rate, and so on. Different data types lead to different data ranges and typical sizes. For instance, usually, the body temperature is within the range 35 °C–45 °C. For a common person, the mean value of the body temperature is 36.9 °C. However, for another kind of health data, such as blood pressure, the data range must be totally different from the body temperature. The maximum of blood pressure should be less than 180 mmHg. In this paper, the maximum of the message should be less than *T*. We only consider the size of the largest message to evaluate the sensitivity and the error of the result. Therefore, the error listed in Table 6 is irrelevant to the type of data. Specifically, once the maximum value and the message amount are both established, we can calculate the mathematical expectation of the square difference of the Laplace mechanism.

**Figure 6:**
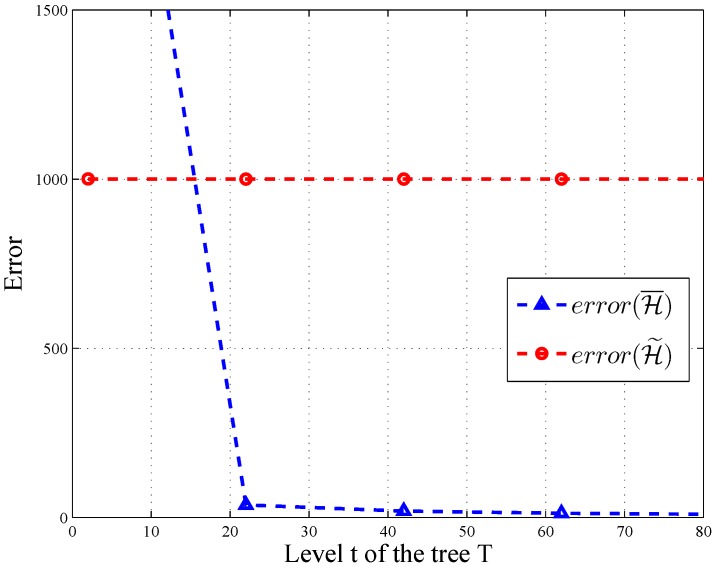
Error varies with the level.

**Figure 7:**
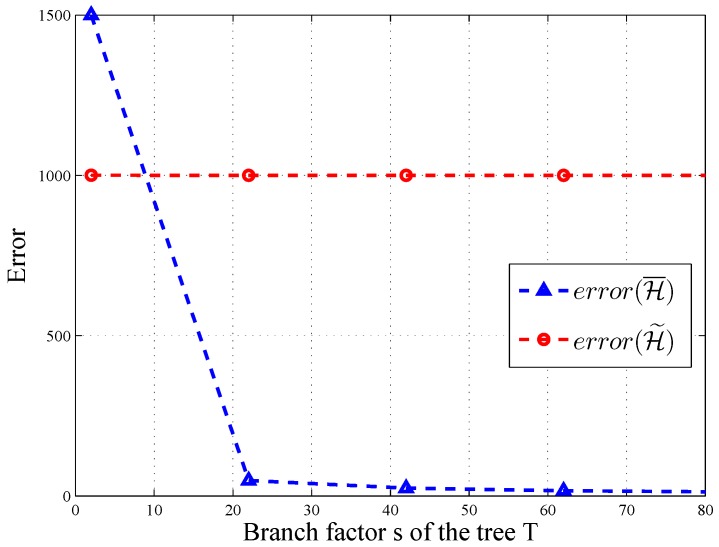
Error varies with the branch.

In HMH, we improve the accuracy of the query through consistency. According to [12], $error\left(\bar{H}\right)=O\left(t^{3}/\epsilon^{2}\right)$ for all query sequences, and there exists a query sequence with $error\left(\bar{H}\right)\leq \frac{3}{2(t-1)(s-1)-s}error\left(\tilde{H}\right)$. As depicted in Figure 6 and Figure 7, the error of $\bar{H}$ changes significantly along with the level and branch of query tree *T*, when ϵ=1.0 and $error(\tilde{H})=1000$. Thus, post-processing makes $\bar{H}$ more accurate on some query sequences.

Another typical way to evaluate the error is to issue queries on the data before and after using differential privacy and measuring the difference between these query results. Therefore, we also give a further analysis of the error by leveraging a popular metric called the relative error [6]. This metric can directly reflect the difference between the original result and the permutated result. The mathematical expectation of the relative error *η* is calculated as follows.

(29) $$\eta =\frac{E|\tilde{M}-M|}{M}=\frac{E|Z|}{M}\;\;\;s.t.\;\;\;Z∼Lap\left(\frac{\Delta f}{\epsilon }\right).$$

where $E|Z|=2\int_{0}^{+\infty }z\cdot Lap\left(\frac{\Delta f}{\epsilon }\right)dz=2\int_{0}^{+\infty }z\cdot \frac{\epsilon }{2\Delta f}\cdot e^{-\frac{z\cdot \epsilon }{\Delta f}}dz=\frac{\Delta f}{\epsilon }$. Therefore, the relative error is $\eta =\frac{\Delta f}{\epsilon \cdot M}$. As the sensitivity of the aggregation functions are already discussed, we can easily calculate *η*. Additionally, the relative errors of each scheme are listed in Table 7.

As analyzed above, the error of WAAS is greater than or equal to that of PAAS and BAAS. Moreover, the errors of ${\mathrm{MHDA}}^{+}$, BAAS and PAAS can be considered equivalent approximately. Here, we assume that the data type is the body temperature. Let ϵ=0.1, *T* = 45 °C (i.e., the maximum temperature of human) and the average temperature *M* = 37 °C. Suppose the number of users is k=10,000. Then, the relative error of BAAS/PAAS is 0.1216%, which is almost the same as $MHDA^{+}$, that is 0.1217%. Therefore, the difference between BAAS/PAAS and $MHDA^{+}$ is negligible.

## 7. Further Discussion

In this section, we will discuss four special issues related to our work.

The first issue is temporal aggregation, which is the aggregation of the same user’s data at different time points. In this paper, we recognize the average aggregation as a representative function of additive aggregation. Specifically, we only give the spatial aggregation schemes, which is the aggregation of the different users at the same time point. Actually, the temporal aggregation is similar to spatial aggregation. The servers can just multiply all of the corresponding user’s encrypted health data to obtain the encrypted sum. Moreover, the jointly decryption of the encrypted sum is exactly the same as the spatial aggregation.

The second issue is fault tolerance. The computational missions are assigned to l+1 CSs in PMHA-DP, and the total number of CSs is more than 2l+1. According to the adversary model, no more than *l* servers could be compromised. Therefore, at least l+1 servers can accomplish computational tasks. Hence, our scheme supports fault tolerance of CS failures. BAAS supports the user failures. When some users refuse to report their health data, the CSs only need to record the number of normal users and calculate the encrypted sum as usual. Then, TA divides the sum by the number of normal users to acquire the average value. Our non-additive schemes also support fault tolerance of user failures. In WAAS, CSs can report the abnormal users’ weights to TA. Then, TA is able to figure out the total weight of the normal users. Thus, the weighted average of normal users can be calculated by TA. Therefore, both WAAS and PAAS support fault tolerance of user failures; because TA can still obtain the normal users’ sum through solving Equation (19). In a word, PMHA-DP achieves some functions and supports fault tolerance simultaneously.

The third issue is data type. PMHA-DP can be extended to some other scenarios with different data types; such as salary, age, height and some other short personal record. As we know, the Boneh–Goh–Nissim cryptosystem applied in this scheme is a homomorphic public key encryption scheme. The same as the other public key cryptosystem, it also suffers from low efficiency of encryption and decryption. Thus, it is often used to encrypt some short messages. Moreover, the proposed solution cannot be adapted to the scenario of file encryption and some other kinds of huge messages. If a user wants to encrypt one’s own video file before outsourcing it to the cloud, the secret key cryptosystem may be a better choice.

The fourth issue is data tampering. The compromised users may send tampered data to the cloud servers. For instance, a malicious user may send some abnormal data to the cloud (e.g., a compromised user may set his or her temperature as 100 °C), which directly impacts the aggregation result. These abnormal data are not easy to detect under the ciphertext environment, and it will indeed decrease the accuracy of the statistics. How to kick out the compromised users and detect the tampered data are both challengeable problems. Several data aggregation schemes [6] are suffering from these problems, as well. At present, we have no practical solution. Therefore, these problems are important, and we will push a further study them in future work.

## 8. Related Work

Cloud-assited WBANs is evolved from the traditional wireless sensor networks (WSNs) [20,21,22], which is widely used in healthcare applications. Actually, some cryptology-based techniques, such as key evolution [23], public verification [24], searchable encryption [25] and user authentication [26], can also be applied in the cloud-assisted WBANs. We will make in-depth study on these techniques in the future. However, in this section, we mainly introduce the state-of-the-art works closely related to our paper. We first review the privacy-preserving data aggregation schemes and then recall some about differential privacy.

Privacy-preserving data aggregation is a kind of cryptology-based technique, which aims at protecting sensitive data. In [8], Lu et al. provide an efficient aggregation scheme called EPPA. It structures multi-dimensional data into a ciphertext by utilizing a super-increasing sequence. Due to the batch verification technique, EPPA significantly decreases the communication and computational overheads. However, the fault tolerance and differential privacy are not supported. In [9], Chen et al. present PDAFT , which supports both spatial and temporal aggregation. Moreover, PDAFT also supports fault tolerance. However, it does not provide multifunctional aggregation and differential privacy guarantees. In [27], Li et al. leverage a novel key management technique to support large plaintext space. Their scheme can accomplish min aggregation, which can be extended to max aggregation. However, it fails to support multifunctional aggregation and fault tolerance. Chen et al. [10] propose a scheme called MuDA . It supports variance aggregation and one-way ANOVA aggregation with differential privacy. Besides, MuDA leads to less communication overhead than the scheme proposed by Shi et al. [28]. However, MuDA cannot support fault tolerance either.

Han et al. [6] present a multifunctional aggregation scheme with fault tolerance called PPM-HDA. PPM-HDA supports both temporal and spatial aggregation. Besides, the differential privacy mechanism is also applied. However, there are still some functions, like weighted average and histogram aggregation, which are not discussed in [6].

Differential privacy [29,30,31] is a promising technique that can achieve a mathematically-precise guarantee of privacy. In [13], Dwork et al. classify data release under differential privacy into two different models: interactive and non-interactive [32,33]. Dwork et al. [13] proposed the widely-used Laplace mechanism.

The private histogram reflects the data distribution. The results can be used for statistical query or other linear queries. Many works are done by using the private histogram. For instance, Blum et al. [32] construct a one-dimensional histogram by dividing the input counts into several bins, where the counts in each bin are approximately equal. Meanwhile, Xiao et al. explore a wavelet-based approach to handle multi-dimensional dataset. In [34], Xu et al. develop several methods of building an optimal histogram under *ϵ*-differential privacy. Multi-dimensional partitioning strategies for differential private histogram release can be found in [35]. Recently, Li et al. [36] proposed a new histogram publish scheme that achieve higher accuracy. However, these schemes are lacking efficiency in the histogram partition and data update.

Some differential privacy mechanisms take post-processing to reduce the noise scale. Boosting the accuracy of the query through consistency is feasible. Barak et al. [37] firstly make a contribution to it. Moreover, Hey et al. [12] answered histogram queries by a basic hierarchical method. They also apply the technique of constrained inference to improve query accuracy. Recently, Lee et al. [38] formulated the post-processing step as a constrained maximum likelihood estimation problem, which is equivalent to constrained $L_{1}$ minimization. The proposed scheme is suitable for a wide variety of applications, including differential private contingency tables, histograms and linear queries.

## 9. Conclusions

In this paper, we propose a privacy-enhanced and multifunctional health data aggregation scheme, which is able to support fault tolerance. Specifically, we utilize the cloud-assisted WBANs system as our storage, computation and communication supporter. The proposed scheme achieves additive and non-additive aggregation simultaneously. We provide a few new aggregation functions and protect the aggregated data from the CSs. The performance evaluation indicates that the communication and computational overhead is reduced. For the future work, we are going to focus on the data aggregation techniques in the environment of big data. We will try to achieve some other functions and boost the efficiency of the scheme.

## Figures and Tables

**Table 1:** Comparison of the functionality of additive aggregation.

	Basic Scheme	Weighted Average	Aggregated Data Protection	Differential Privacy
${\mathrm{MHDA}}^{+}$[6]	*√*	×	×	*√*
PMHA-DP	*√*	*√*	*√*	*√*

**Table 2:** Comparison of the functionality of non-additive aggregation.

	Max/Min	Median	Hierarchical Method	Post-Processing	Differential Privacy
${\mathrm{MHDA}}^{⊕}$ [6]	*√*	*√*	×	×	*√*
PMHA-DP	*√*	*√*	*√*	*√*	*√*

**Table 3:** Notations.

Symbols	Meanings
$T_{exp}$	time of modular exponential calculation in $Z_{n^{2}}$
$T_{mul}$	time of modular multiplication
$T_{bim}$	time of bilinear map operation
$T_{pol}$	time of using Pollard’s lambda method to compute the discrete logarithm
*k*	the number of mobile users
l+1	the number of working cloud servers

**Table 4:** Comparison of the computational overhead. BAAS, basic average aggregation scheme; WAAS, weighted average aggregation scheme; PAAS, privacy-enhanced aggregation scheme; MU, mobile user; CS, cloud server; TA, trusted authority.

	MU	SP	CSs	TA
BAAS	$2T_{exp}+T_{mul}$	N/A	$\left(l+1\right)T_{exp}+\left(k+l-1\right)T_{mul}+T_{pol}$	N/A
WAAS	$2T_{exp}+T_{mul}$	N/A	$\left(l+1\right)T_{exp}+\left(k+l-1\right)T_{mul}+kT_{bim}+T_{pol}$	$2kT_{exp}+kT_{mul}$
PAAS	$6T_{exp}+3T_{mul}+2T_{bim}$	N/A	$2\left(l+1\right)T_{exp}+2\left(k+l-1\right)T_{mul}+2T_{pol}$	N/A
${\mathrm{MHDA}}^{+}$ [6]	$2T_{exp}+T_{mul}$	$\left(k-1\right)T_{mul}$	$\left(l+1\right)T_{exp}+lT_{mul}+T_{pol}$	N/A

**Table 5:** Comparison of the error.

	BAAS	PAAS	WAAS	HMH	${\mathrm{MHDA}}^{+}$
error	$\frac{2T^{2}}{\epsilon^{2}{\left(k-1\right)}^{2}}$	$\frac{2T^{2}}{\epsilon^{2}{\left(k-1\right)}^{2}}$	$\frac{2T^{2}w_{max}^{2}}{\epsilon^{2}{\left(\sum_{weight}-w_{max}\right)}^{2}}$	$\frac{2mt^{2}}{\epsilon^{2}}$	$\frac{2e^{\frac{k\epsilon }{T}}}{e^{2\frac{k\epsilon }{T}}-2e^{\frac{k\epsilon }{T}}+1}$

**Table 6:** List of error values.

*w*	12	13	14	15	16	17	18	19	20	21
*k*	10,000	20,000	30,000	40,000	50,000	60,000	70,000	80,000	90,000	100,000
BAAS/PAAS	33.54	33.55	59.65	134.21	343.59	954.42	2804.86	8589.90	27148.38	87960.85
${\mathrm{MHDA}}^{+}$	33.37	33.38	59.48	134.04	343.42	954.26	2804.69	8589.75	27148.25	87960.80

**Table 7:** Comparison of the relative error.

	BAAS	PAAS	WAAS	${\mathrm{MHDA}}^{+}$
relativeerror	$\frac{T}{(k-1)M\epsilon }$	$\frac{T}{(k-1)M\epsilon }$	$\frac{Tw_{max}}{(\sum_{weight}-w_{max})M\epsilon }$	$\frac{2e^{-\frac{k\epsilon }{T}}}{M(1-e^{-\frac{2k\epsilon }{T}})}$
